# COVID-19 Research: Challenges to Interpret Numbers and Propose Solutions

**DOI:** 10.3389/fpubh.2021.651089

**Published:** 2021-04-12

**Authors:** Marc J. Struelens, Paolo Vineis

**Affiliations:** ^1^Faculty of Medicine, Universite Libre de Bruxelles, Brussels, Belgium; ^2^School of Public Health, Imperial College, London, United Kingdom

**Keywords:** COVID-19, epidemiologic methods, transmission models, surveillance, open science, pandemic control, evidence-based policy, researcher responsibility

## Abstract

The response of the scientific community to the COVID-19 pandemic has been unprecedented in size, speed and discovery output. Within months of virus emergence, the SARS-CoV-2 genomics, replication, evolution and dissemination dynamics as well as natural history, infection risk and prognostic factors and biology of the disease have been gradually deciphered. More than 250 articles on COVID-19 published in Frontiers in Public Health have contributed to these insights. We discuss here some of the key research themes and challenges that have been addressed. We provide our perspective on current research issues with surveillance data quality and limitations of epidemiological methods. We warn against the potential misuse or misleading interpretation of public data of variable quality and the use of inadequate study designs for the evaluation of effect of non-pharmaceutical interventions. We conclude by interrogating possible public health strategies for pandemic control as well as discuss the ethical responsibilities and democratic accountability of researchers in their role as experts and policy advisors.

## The outstanding research response to the pandemic

The COVID-19 pandemic has inflicted immense health and societal damage across the world in 2020, with over 1.9 million deaths, massive economic recession and life disruption related to drastic social distancing and other control measures. In comparison to any other infectious disease, the response of the scientific community has been unprecedented in size, speed and discovery output. Within months of COVID-19 emergence, the SARS-CoV-2 genomics, replication, evolution and dissemination dynamics as well as natural history, infection risk and prognostic factors, biology and pathogenesis of the disease have been gradually deciphered. Novel diagnostic approaches and assays have been developed, while an array of antiviral and immunomodulating agents were tested in multicentre trials. COVID-19 vaccines have been developed at record speed with several safe and effective products authorized and launched for mass immunization in December 2020.

To make sense of emerging information on this pandemic, one should acknowledge how these extraordinary research efforts have generated an explosion of scientific papers. Bibliometric surveys indicate that more than 180,000 peer-reviewed articles and 30,000 preprints on COVID-19 were published this year (1). This surge has required substantial time and resource investment from authors, editors and publishers. From the outset of the pandemic, *Frontiers* has facilitated the timely peer-review and open-access publication to date of 2,000 scientific papers in 168 COVID-19 dedicated Research Topics across its journals (2). In *Frontiers in Public Health*, manuscript submissions on infectious diseases have increased 5-fold this year as compared with last year, 80% of which report on COVID-19 research. We wish to thank our authors, editors and reviewers for their time and commitment to deliver over 250 articles advancing our understanding of COVID-19-related issues. We discuss here some of the key research themes that have been addressed in 2020 in *Frontiers in Public Health*, with selected examples of salient contributions from this Journal.

## Marked Geographical Heterogeneity in COVID-19 Burden

One of the striking public health features is that countries around the world are facing such dramatically different impact of the pandemic both on morbidity and mortality. Though different health burden indicators have different meanings, time dependencies and pitfalls, they consistently indicate that some countries fare much worse than others. As of 21 December 2020, the continuously increasing cumulative COVID-19 incidence ranged by country worldwide from 3.26 to 98,388 total confirmed cases per million population (3) while COVID-19 related mortality ranged by country worldwide from 0.03 to 163 death per 100,000 population (4). As indicator of lethality, the crude case-fatality (CF) among notified COVID-19 cases ranged at latest report from 0 to 29%, with many high-income countries falling in the higher range (4). Likewise, modeled estimates of SARS-CoV-2 infection fatality (IF) inferred from population sero-surveys in 11 selected countries and cities range from 0.14 to 0.42% in low-income countries to 0.78–1.79% in high income countries, with the differences in those ranges related to the older population of high-income countries (5). Another mortality indicator, the number of excess deaths from all causes above historical baseline that are temporally associated with COVID-19 incidence peaks, does not depend on diagnostic testing nor disease reporting capacity. Data from countries that monitor this more robust indicator confirm their contrasting mortality burden and reveal further geographical heterogeneity within the larger ones (6, 7). These data also highlight striking variation in the degree of national underreporting of COVID-19-related deaths (7). Many published data illustrate the heterogeneity of COVID-19 epidemiologic indicators, that complicates our understanding of the heterogeneity of the burden across countries.

## Use and Misuse of Epidemiologic Surveillance Data

Extensive epidemiologic and multidisciplinary research has explored possible determinants to these major variations in the pandemic burden between countries and regions. These studies have taken advantage of notification of COVID-19 surveillance data by national authorities and of open data compilation and trends monitoring by academic centers (3, 4) and supranational agencies like the WHO and ECDC. However, the first step that is often overlooked by researchers in international comparative studies is appraisal of the accuracy and comparability of national surveillance statistics. Unfortunately, national COVID-19 surveillance methods are not standardized and their implementation further depends on local clinical and laboratory capabilities and practice. Differences and changes over time in national surveillance protocols, case definitions and reporting delays in the European Union are monitored by ECDC (8). Inter-country variation affects the following steps of surveillance data reporting: 1. case definition criteria, such as for the attributed cause of death (laboratory confirmed COVID-19 case only or also probable case, time delay of death after positive COVID-19 test, type of test - PCR, antigen test, serology, place of death - in hospital or also community deaths); 2. case ascertainment (SARS-CoV-2 testing policy, testing rate, testing method quality); 3. case notification delays and corrections. There is a certain degree of confusion around commonly used metrics, and clarifications are needed. A good introduction to the methodological issues and main types of biases behind apparent inter-country differences in pandemic health outcomes is discussed by Backhaus (9). Specific warnings against misinterpretation of COVID-19 surveillance data and their derived indicators are provided in public health and academic resources (3, 8). To enhance preparedness and support response to this and future pandemics, national infectious diseases surveillance systems should further standardize and improve the collation and timely reporting of complete, disaggregated and comparable epidemiologic data.

Ecological studies on potential determinants of COVID-19 outcomes across countries have used open data on environmental conditions, population demographics, economic resources, health systems, and public health policies. Some of these health determinants are conveniently summarized in composite indices such as those provided by the Oxford COVID-19 Government Response Tracker initiative, aimed at comparing the stringency of government responses to COVID-19 across countries and over time (10). Attempting to use such indices to address the effectiveness of epidemic containment policies and interventions raises a number of methodological caveats (3, 10, 11). Articles, including from this Journal, are open to questioning as to the study design, quality of data and interpretation. One example is the report by De Larochelambert et al. (12), that explored five domains (demography, public health, economy, policy, environment) and their potential associations with COVID-19 mortality during the first 8 months of 2020, through a Principal Component Analysis and Pearson correlation tests. Although it raises interesting points about the background weaknesses of the countries that were more affected by the early phase of the pandemic, most of these features are correlated with an aging population. The pitfalls with the study include a lack of age-standardization of death rates, and a lack of consideration of the timing of and compliance with public health interventions. First, COVID-19 CF and IF steeply increase with age above 50 years (3–5). Comparing the COVID-19 related risk of death in populations with different age structures by age- standardized mortality as attempted by Villani et al. (13), shows very different ranking of countries than by using crude mortality data. Second, the analysis of public health interventions through national scores of the Oxford University Containment and health index and Stringency index, as predictors of COVID-19 mortality in this study is in our opinion inadequate as neither the degree of policy implementation, nor the timing of interventions in their epidemiological context were taken into account. Indeed, the Oxford University government index designers warn that “these indices should not be interpreted as a measure of the appropriateness or effectiveness of a government's response” (10). Time series and temporally weighted regression analysis of these indices indicate that high stringency control policies may be associated with divergent trends in national mortality depending on whether it was initiated early or late after the start of the epidemic (10, 11). Therefore, the conclusions by De Larochelambert et al. (12) that the “stringency of the measures settled to fight pandemia (sic), including lockdown, did not appear to be linked with death rate” and that “this (mortality) burden was not alleviated by more stringent public decisions” are simply meaningless. Unfortunately, this publication has been cited by conspiracy theorists on social media as supporting claims that “Lockdowns do not control the coronavirus: The evidence” (12).

This is only an example of the caution that needs to be exerted before drawing inferences from open data that are of variable quality and sometimes wrongly interpreted. On the other hand, epidemiologic studies reported in this journal have progressed our understanding of the risk factors associated with SARS-CoV-2 transmission and COVID-19 fatal outcomes (13–19). Likewise, modeling studies have shed light on effective control measures and likely trajectory of the pandemic in various settings (20–23).

On risk factors, an early case series by Jin et al. on the role of gender in morbidity and mortality in patients with COVID-19 showed that men are at higher risk for severe disease and death, independent of age (14). Li et al. analyzed the COVID-19 incidence and mortality risk in China using a maximum likelihood approach that indicated a steeply increasing mortality risk in older adults (15). In an ecological study, Khan et al. used a negative binomial regression model and Principal Component Analysis to assess the association between national healthcare capacity index (number of physicians, nurse and hospital beds per population) and crude COVID-19 CF data available on 30 April 2020 from 86 countries, adjusting for other covariates (demographic, health expenditure, population density, and prior burden of non-communicable disease as well as civil society openness index) (16). While acknowledging the data limitations and possible biases, their analysis confirmed that greater healthcare capacity was related to lower COVID-19 CF (16). This has been experienced very acutely in countries confronted with insufficient intensive care capacities for managing a surge of patients with respiratory failure during epidemic peaks (6, 8, 13, 16).

More detailed epidemiologic investigations revealed important determinants of epidemic spread at the local level by using diverse statistical models (17, 18). De Ridder et al. used a spatiotemporal cluster detection algorithm to monitor SARS-CoV-2 transmission dynamics in neighborhoods of Geneva until 30 April 2020 (17). By using survival analysis and Cox model adjusted for population density, they found a dose-response relationship between level of socio-economic deprivation and prolonged duration of virus transmission within local clusters, highlighting the need for inequality mitigation measures as part of COVID-19 risk mitigation strategies. Castaneda and Saygili examined the county-level proportion of residents staying at home as measured by mobile device location data and COVID-19 daily case increase rates in Texas during February-May 2020 (18). They found that the growth rate of COVID-19 cases decreased when a larger proportion of the local population stayed at home. Interestingly, county emergency policies coincided more closely with the increase of people at home than the later State-wide order to “shelter in place,” suggesting that to reach out with an alert to the local population may be a more effective communication strategy in that setting (18). Bönisch et al. assessed the effect of confinement in Germany by using an Interrupted Time-Series analysis linking actively collected population mobility data and weekly estimates of COVID-19 reproduction number before, during and after the lockdown from January to May 2020 (19). They measured a significant mobility decrease by more than half across all age groups and regions during lockdown and to a lesser degree thereafter. This mobility reduction was followed after a few weeks by a sustained reduction in COVID-19 transmission as indicated by an effective reproduction number falling from a value of ~3 to below 1 (19).

Mathematical modeling has been extensively applied during this pandemic to nowcast and forecast its national trajectory and impact on healthcare resources and to inform decisions about control interventions (20, 21). It is important to remember that all models are a simplified hypothetical representation of reality. In a helpful commentary for the non-mathematician, Mac et al. clearly explain the main types of epidemic models used for analyzing and projecting SARS-CoV-2 transmission and discuss their respective strengths and limitations (21). The authors underline the advantages of defining the modeling questions, appraising the input data quality and model assumptions in partnership with stakeholders who work in the field and may use the results for making practical decisions (21).

A range of mechanistic models formalize the transition of groups or individuals in a population from the susceptible to infected to recovered (SIR) states, with model variation by inclusion of further states such as death (SIRD) or susceptible again (SIRS) (21). In Frontiers in Medicine, Roques et al. applied an elaborate analytical framework combining a SIRD transmission model, a probabilistic observation model and Bayesian inference procedure to measure the effect of the nationwide lockdown in France in March 2020 (22). They estimated that the lockdown effectively reduced the transmission of the COVID-19 by a factor 7, based on an effective reproduction number R_e_ = 0.47 during lockdown compared to the basic reproduction number R_0_ = 3.2 in the early stage of the epidemic (22). With yet another approach, Wang et al. developed a survival convolution model to fit the dynamics of national epidemics and estimated the effect of nationwide control interventions in selected countries through a natural quasi-experimental design (23). Their forecasting results predicted better COVID-19 transmission control in China and Korea than in Italy and the USA after relaxing restriction measures in the spring 2020 (23).

Overall, as illustrated above, idiosyncrasies abound in COVID-19 reported data, and competing analytical approaches do not allow easy interpretations of observations across settings. Therefore, one should consider their limited external validity to infer actions, especially when attempting to generalize effectiveness beyond the local or national context. We acknowledge that relying only on studies published in *Frontiers* is a limitation of the wider perspective that could come from reviewing publications in other Journals on COVID-19 epidemiology and control.

## Prospects for the Future

Frankly, and as consequence of the above warnings and limitations of the assessed evidence, it is not clear what the optimal strategy for the future control of the COVID-19 pandemic is beyond mass vaccination. There are great expectations toward the vaccines, several of which have been recently authorized for emergency use or approved by regulators after demonstrating high levels of efficacy and safety in trials. Their administration has started in many countries in December 2020. The first real-world estimates of very high levels of short-term protective effectiveness against infection and disease from national vaccination campaigns are extremely encouraging (24). However, uncertainties remain about the duration of vaccine-induced protection against asymptomatic infection and against disease in vaccinated individuals, especially in the event of emergence and spread of viral antigen-variants that escape vaccine-induced immunity. The ultimate goal of reaching herd immunity across populations will require extensive immunization campaigns and wide population coverage, and will likely occur later than 2021.

Therefore, a multipronged strategy of testing, tracing and isolating infectious cases and their contacts, in combination with sustainable levels of social distancing and use of personal protection such as face masks, remains the best option beyond vaccination to reduce viral transmission to minimum levels (25). In many countries, experience has shown that molecular testing for SARS-CoV-2 RNA has limited capacity related to the laboratory resources available and the delays in obtaining results. On this basis, decentralized rapid antigen detection tests have acquired considerable popularity. Their optimal use has been outlined in a clear way by the ECDC (26) and OECD (27), i.e., the test can be useful for testing recent contacts of cases or screening particular categories of subjects, such as elderly people living in closed communities and health sector workers. In spite of these guidelines, people in several countries may access the rapid test e.g., through pharmacies, and test themselves with no consultation with GPs or coordination with preventive services. This approach has several drawbacks. In particular, the antigen tests have limited sensitivity, around 70% but ranging from 20 to 95%, while specificity is higher (26, 27). False positives can be ruled out by confirming positive antigen test results with a nucleic acid test. Even though the best antigen tests would detect a majority of infected people with a high viral load who are likely to be the most infectious, a negative result on a given day does not predict non-infectiousness thereafter. In a period of sustained community-wide transmission of SARS-CoV-2, there is a risk of false security if people perform the rapid antigen test to allow themselves participation in group gatherings and festive activities, loosening their observance of preventive measures and thereby acquiring and spreading the virus further.

An experiment of population-wide screening for SARS-CoV-2 infection with rapid antigen tests has been performed in Slovakia but the evaluation of its impact is complicated by combination with lockdown measures (28). A one-off cross-sectional screening campaign is likely not enough to isolate all the infected individuals and quench the epidemic. It is not clear what follows after such a complex and expensive testing experiment. Further research to assess the cost-effectiveness of this approach is to be encouraged. In addition, extensive genomic surveillance and structure-function molecular studies are essential to monitor the possibility of emergence of diagnostic or vaccine escape viral variants with mutations in the antigen-encoding genes (29).

Biomedical and public health experts are playing a prominent and essential role around the world in providing evidence-based advice to the public and government on measures to suppress or slow down COVID-19 spread, with varying degree of decision-making responsibility. At the same time, increasingly large parts of the public have been expressing distrust of expert knowledge and reclaiming their autonomy of decision from technocratic policies, as illustrated in the anti-vaccination movement (30). In this Journal, Lavazza and Farina opine that expert recommendations on risk management such as priority access to testing or intensive care, or digital tracing of personal contacts, are not neutral and carry axiomatic content that goes beyond the epistemic authority of scientific experts (30). We support their view that decisions which are not only technical but also normative must be justified as such and subject to wider participatory democratic decision-making. In the line of the above discussion about the limited robustness of available epidemiologic data, transparency about uncertainty in scientific inference based on the analysis of these data is a moral imperative. As underlined by Provenzi and Barello, building trust between lay citizens and researchers on identifying COVID-19 solutions also requires a renewed partnership that includes public education on the scientific method as well as the active participation of “citizen scientists” in biomedical investigations and health intervention trials (31).

## Data Availability Statement

The original contributions presented in the study are included in the article/supplementary material, further inquiries can be directed to the corresponding author/s.

## Author Contributions

All authors listed have made a substantial, direct and intellectual contribution to the work, and approved it for publication.

## Conflict of Interest

The authors declare that the research was conducted in the absence of any commercial or financial relationships that could be construed as a potential conflict of interest.
